# A Predictive Model and Comparative Analysis of Laser-Induced Phase Transition Thresholds for Four Key Engineering Alloys

**DOI:** 10.3390/ma19050927

**Published:** 2026-02-28

**Authors:** Lyubomir Lazov, Lyubomir Linkov, Nikolay Angelov, Edmunds Sprudzs, Arturs Abolins

**Affiliations:** Rezekne Academy, Riga Technical University, Atbrivosanas Aleja 115, LV-4601 Rezekne, Latvia; lyubomir.linkov@edu.rtu.lv (L.L.); angelov_np@abv.bg (N.A.); edmunds.sprudzs@rtu.lv (E.S.); arturs.abolins_1@rtu.lv (A.A.)

**Keywords:** laser technologies, critical surface power density for melting, critical surface power density for evaporation, scanning speed, process window, titanium, C26000 brass, SS304 stainless steel, 42CrMo4 alloy steel

## Abstract

Laser-based manufacturing processes—including marking, hardening, cutting, and welding—demand the precise selection of processing parameters, as the resulting surface state is critically dependent on the delivered power density and beam–material interaction time. This study presents a unified predictive framework for estimating the critical surface power density thresholds for melting *q_scm_* and evaporation *q_scv_* as functions of scanning speed *v* for the following four technologically important metallic materials: titanium, C26000 brass, SS304 stainless steel, and 42CrMo4 alloy steel. The principal novelty of this work is twofold. First, it provides the first directly comparative analysis of these four materials under identical, standardized laser conditions (*λ* = 1064 nm, *d* = 40 μm, constant absorptivity *A* = 0.4), eliminating the confounding effects of variable beam geometries and optical assumptions that hinder cross-study comparisons. Second, it translates fundamental thermophysical principles into a practical engineering tool, such as a validated spreadsheet calculator that outputs material-specific threshold curves in real time, enabling rapid, physics-based parameter estimation without recourse to complex numerical simulations. The computed threshold curves exhibit a consistent non-linear increase with scanning speed for all materials, governed by the inverse relationship between interaction time and required power density. The following clear material hierarchy emerges: C26000 brass exhibits the highest thresholds (e.g., *q_scm_* = 0.94 × 10^10^ W/m^2^, *q_scv_* = 10.74 × 10^10^ W/m^2^ at *v* = 100 mm/s) due to its high thermal conductivity, while titanium shows the lowest (*q_scm_* = 0.19 × 10^10^ W/m^2^, *q_scv_* = 0.48 × 10^10^ W/m^2^ at *v* = 100 mm/s) as a consequence of strong heat confinement. SS304 and 42CrMo4 occupy intermediate positions, with 42CrMo4 demonstrating notably higher evaporation resistance than SS304 despite similar melting thresholds. The resulting dual-threshold framework delineates three distinct process regimes—sub-melting heating, melting-dominant processing, and evaporation—providing a quantitative basis for parameter selection in applications ranging from surface hardening to micromachining. By bridging the gap between theoretical material science and applied manufacturing, this work offers a robust, first-order reference for process design and establishes a methodological template for future comparative studies of laser–material interactions.

## 1. Introduction

The laser processing of materials has become a central technology in modern manufacturing due to its precision, flexibility and ability to process a wide range of materials, including surface characteristics, with controlled energy input [1,2]. In processes such as texturing, marking, hardening, cutting, welding, etc., the quality of technological processes is determined primarily by the power density of the laser surface and the duration of interaction between the laser beam and the material surface [3,4]. In laser processing, in addition to these two main factors, the duration of the laser pulses, frequency, scan step, etc., also have a significant impact, which makes the choice of these parameters critical for ensuring the desired results, such as selective oxidation, melting or ablation [5,6,7]. Laser–material interaction is a complex thermophysical phenomenon, in which the absorbed laser electromagnetic energy causes localized heating and the subsequent process of heat conduction in depth and in the radial direction from the absorption zone. The interaction regime is determined by the balance between the absorbed electromagnetic laser energy, the heat diffusion, and the specific thermophysical properties of the target material, including melting and vaporization temperatures, as well as the latent heats of melting and vaporization [8,9,10]. Heating the surface below the melting point can change the surface microstructure without a phase change, often associated with a surface oxidation process, while sufficient surface power density can lead to melting and, at even higher energy densities, vaporization [11,12,13]. A key parameter in characterizing these regimes is the critical surface power density threshold for phase transitions. The melting threshold corresponds to the minimum energy per unit area required to raise the surface to the melting point, while the evaporation threshold requires overcoming a significantly higher energy density to reach boiling conditions and provide latent heat of vaporization [14,15,16]. The difference between these thresholds is essential and fundamental for determining the operating windows for different laser processes. Understanding how these thresholds vary with scanning speed and material properties is essential for reliable process design and optimization. The scanning speed affects the dwell time of the laser beam at a given location on the surface; higher scanning speeds reduce the dwell time in the absorption zone and, therefore, the energy delivered per unit area per unit time, thus requiring a higher power density at the incident surface to achieve the same phase change criterion [5,17]. This leads to a characteristic nonlinear dependence of both melting and vaporization thresholds on the scanning speed, which has been observed in a number of experimental and model studies in laser manufacturing and additive processes [6,18,19]. Although many scientific research and experimental works have investigated these phenomena and dependencies, the methods of numerical modeling and simulations of the behavior of laser melting and ablation for specific materials or conditions of specific technological processes remain limited. This also applies to the unified comparative assessment of melting and vaporization thresholds for certain metals and alloys under the conditions of different laser technological treatments. Overcoming this gap not only provides a deeper insight into the dependence of the specific physical characteristics of the processed material on the laser interaction, but it also supports the development of practical technological windows that outline effective operating modes and, ultimately, the quality of the technological operation [20,21].

While the theoretical dependence of phase transition thresholds on scanning speed is well-established in principle, we lack a practical, directly comparative tool for engineers. Published data often focus on single materials, use varying laser parameters (wavelength, spot size), or are embedded in complex numerical models, making cross-material comparison and preliminary parameter estimation difficult. This work bridges this gap by implementing a unified analytical model under standardized conditions (*λ* = 1064 nm, *d* = 40 µm, *A* = 0.4) to generate a directly comparable dataset of *q_scm_* and *q_scv_* for Ti, C26000 brass, SS304, and 42CrMo4. The primary novelty lies not in the model itself, but in its systematic application to create a validated predictive framework and a comparative ranking based on fundamental thermophysical properties. The resulting threshold curves and the accompanying spreadsheet tool provide immediate, first-order guidance for process design, defining safe operating windows to avoid undesirable regimes such as insufficient heating or excessive ablation. In this work, we present a preliminary theoretical-engineering assessment of the critical surface power density thresholds for surface melting *q_scm_* and evaporation *q_scv_* as functions of scan rate for the following four technologically relevant metallic materials: titanium, C26000 brass, SS304 stainless steel, and 42CrMo4 alloy steel. By using a unified set of thermophysical and optical input parameters, the calculated threshold curves aim to make a comparison between different materials to clarify the influence of thermophysical heat transfer, phase transition requirements, and interaction time on the threshold behavior. Numerical model calculations aim to establish the possible process windows, which is crucial for the rational choice of parameters in laser processing of materials, and motivate subsequent experimental validations.

## 2. Materials and Methods

The analysis considers the following four technologically relevant metallic materials: titanium, C26000 brass, SS304 stainless steel, and 42CrMo4 alloy steel. These materials represent a broad range of industrially relevant alloys with diverse thermophysical properties and are the focus of ongoing experimental research at the Laser Center of RTU Rezekne Academy, Rezekne, Latvia, involving graduate and PhD students. The model is designed to be extensible to other materials of industrial importance by simply updating the input parameters in Table 1.

The optical and thermophysical input parameters required for threshold estimation of surface melting and evaporation power density—including effective surface absorptivity *A*, thermal conductivity *k*, density *ρ*, specific heat capacity *c*, thermal diffusivity *a*, melting temperature *T_m_*, vaporization temperature *T_v_*, latent heat of melting *L_m_*, and latent heat of evaporation *L_v_*—were compiled from authoritative, peer-reviewed sources.

Regarding primary sources, baseline values for density, specific heat, melting temperature, and latent heats were obtained from Mills [22], the standard reference for thermophysical properties of commercial alloys. Thermal conductivity and thermal diffusivity values were cross-referenced against the TPRC Data Series [23] and the compilation by Touloukian et al. [24]. Vaporization temperatures and corresponding latent heats of evaporation were sourced from the NIST Chemistry WebBook [25] and supplemented with data from Iida and Guthrie [26] for molten metals.

For verification and consistency checking, where multiple values existed in the literature (e.g., for thermal conductivity of SS304), we adopted the most frequently cited value and verified its consistency with the derived thermal diffusivity via the relation *a = k*/(*ρc*). For C26000 brass, property values were further validated against manufacturer data sheets (MatWeb [27]) to ensure industrial relevance. For the present first-order engineering assessment, all properties are treated as temperature-independent; this simplifying assumption is explicitly addressed in the Discussion section (Section 4.2).

The model corresponds to scanning irradiation by a continuous-wave fiber laser with wavelength *λ* = 1064 nm and a circular work-spot of diameter *d* = 40 μm on the workpiece surface. Laser absorption is represented through an effective surface absorptivity *A*, which in this study is taken as a constant *A* = 0.4 for all four materials. This value is characteristic of non-polished metallic surfaces at 1064 nm and is adopted here in order to suppress wavelength- and roughness-dependent variations and to emphasize the influence of thermophysical properties and phase-transition enthalpies on the threshold behavior. Under these assumptions, energy is absorbed in a thin near-surface layer and subsequently redistributed into the bulk by heat conduction. The threshold surface power densities for melting and evaporation are obtained from a one-dimensional, conduction-controlled moving-source model. The critical surface power density for the onset of melting, *q_scm_*, and for the onset of evaporation, *q_scv_*, can be written as follows:

(1) $$q_{scm}=\frac{\left(1+s)\;k\;(T_{m}-T_{0}\right)}{2A}\sqrt{\frac{\pi v}{ad}},$$

(2) $$q_{scv}=\frac{\left(1+s^{\prime })\;k\;(T_{v}-T_{0}\right)}{2A}\sqrt{\frac{\pi v}{ad}},$$

where the dimensionless energy storage parameters *s* and *s*′ are defined as follows:

(3) $$s=\frac{L_{m}}{c\;(T_{m}-T_{0})},$$

(4) $$s^{\prime }=\frac{L_{m}+L_{v}}{c\;(T_{v}-T_{0})}.$$

In Equations (1)–(4), the quantities *k*, *a*, *c*, *T_m_*, *T_v_*, *L_m_*, *L_v_*, and *T*_0_ are the material parameters listed in Table 1, whereas *v* denotes the scanning speed. These relations couple conduction-controlled heat flow in the bulk with the finite interaction time defined by the motion of the laser beam. For a scanning beam with constant spot diameter *d*, the local interaction time can be expressed as follows:

(5) $$\begin{array}{cccc} & t_{int}=\frac{d}{v}, & & \end{array}$$

so that increasing the scanning speed reduces the residence time of the beam on a given surface element and, therefore, requires higher incident surface power density to reach the same phase-transition criterion.

For the chosen spot diameter *d* = 40 μm, the local interaction time decreases from *t_int_* ≈ 4 ms at *v* = 10 mm/s to about 0.4 ms at *v* = 100 mm/s, i.e., by one order of magnitude. In the present numerical experiment, Equations (1)–(5) were evaluated for scanning speeds in the range *v* = 10–100 mm/s. This interval is representative of continuous-wave laser texturing and marking systems and provides a realistic basis for comparing the four materials under identical optical conditions. All materials listed in Table 1 were analyzed under the same beam geometry *d* = 40 μm and the same absorptivity *A* = 0.4, which ensures direct comparability of the resulting threshold curves.

The thermophysical and optical parameters used in the calculations are summarized in Table 1.

## 3. Results

### 3.1. Individual Threshold Curves per Material

**Figure 1:**
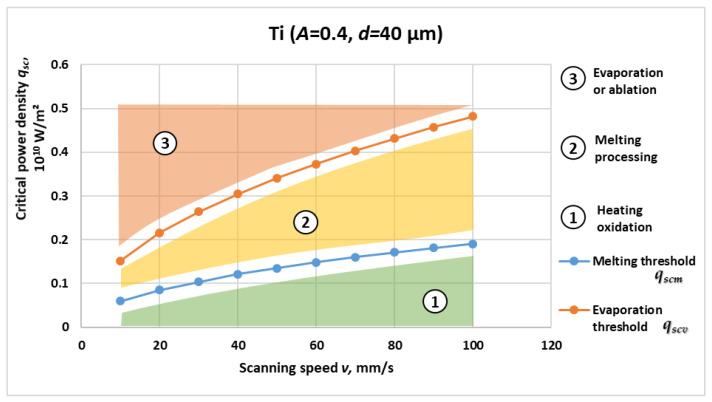
Critical power density for melting *q_scm_* and evaporation *q_scv_* versus scanning speed for titanium.

**Figure 2:**
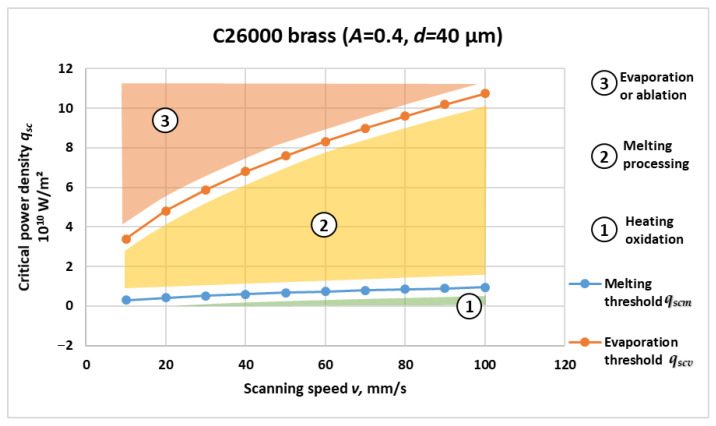
Critical power density for melting *q_scm_* and evaporation *q_scv_* versus scanning speed for C26000 brass.

**Figure 3:**
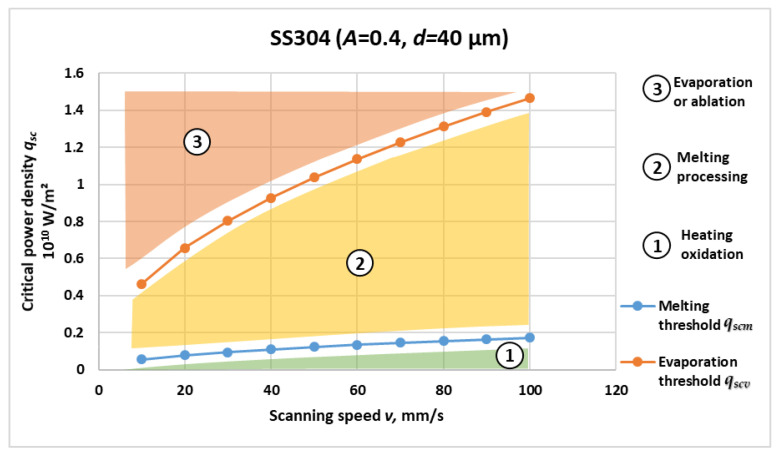
Critical power density for melting *q_scm_* and evaporation *q_scv_* versus scanning speed for SS304 stainless steel.

**Figure 4:**
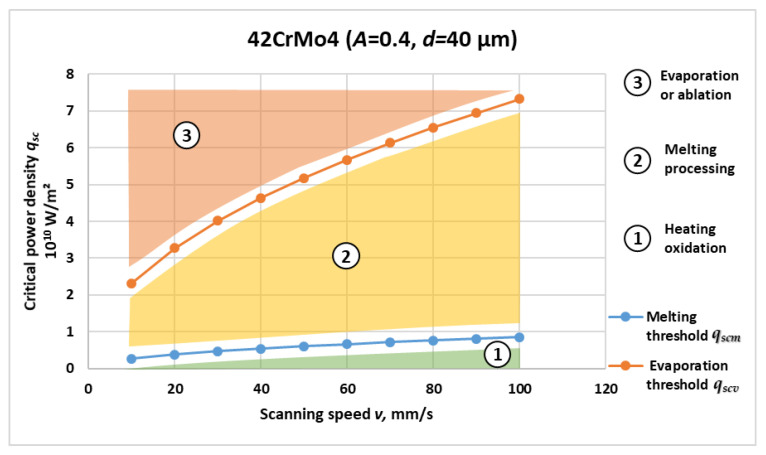
Critical power density for melting *q_scm_* and evaporation *q_scv_* threshold versus scanning speed for 42CrMo4 steel.

Figure 1, Figure 2, Figure 3 and Figure 4 present the computed critical power density for melting and evaporation for each material. For all four materials, both thresholds increase non-linearly with scanning speed, reflecting the reduced interaction time at higher *v* and the higher incident power density required to reach phase-transition conditions. The two-threshold representation provides a compact basis for separating operating regimes in process-window selection.

Based on the graphical dependencies in Figure 1, Figure 2, Figure 3 and Figure 4, the following three distinct areas are clearly outlined for laser interaction with the studied materials:

- Area 1: Located below the critical power density for melting versus speed graph. In this region, laser marking is performed by oxidation, and the temperature is lower than the melting temperature of the material.
- Area 2: Located between the critical power density for melting and the critical power density for evaporation. The laser marking process by melting is implemented here. The material temperature in this region is higher than the melting temperature and lower than the evaporation temperature.
- Area 3: Located above the critical power density for evaporation versus speed graph. The laser marking process by evaporation is implemented here. The material temperature in this area is higher than the evaporation temperature.

Figure 1 presents the calculated critical power density for the melting *q_scm_* and evaporation *q_scv_* of titanium as functions of scanning speed. Both thresholds exhibit a clear monotonic, non-linear increase with scanning velocity, characteristic of conduction-limited laser–metal interaction regimes. At lower scanning speeds of 10–30 mm/s, the beam residence time per unit area is relatively long, 4–1.33 ms for *d* = 40 μm, allowing efficient heat accumulation and thus requiring comparatively lower incident power densities to reach phase-transition temperatures. As the scanning speed increases towards 100 mm/s, the interaction time decreases to about 0.4 ms, so that a smaller fraction of the incident energy is absorbed within a given surface element and proportionally higher power densities are needed to achieve melting or evaporation conditions.

Quantitatively, the melting threshold *q_scm_* rises from approximately 0.06 × 10^10^ W/m^2^ at 10 mm/s to about 0.19 × 10^10^ W/m^2^ at 100 mm/s. The evaporation threshold *q_scv_* follows the same trend, increasing from roughly 0.15 × 10^10^ W/m^2^ to 0.48 × 10^10^ W/m^2^ at 100 mm/s. The ratio *q_scv_*/*q_scm_* remains nearly constant over the full scanning-speed range and is of the order of 2.5–3, which is consistent with the thermodynamic disparity between the melting and vaporization enthalpies of Ti, where the latent heat of evaporation is almost 27 times greater than that of melting.

From a physical standpoint, titanium demonstrates relatively low thermal conductivity ≈22 W/m·K and moderate heat capacity ≈520 J/kg·K, resulting in pronounced heat confinement near the irradiated surface. This explains both the curvature of the threshold curves and their sensitivity to scanning speed, since reduced heat diffusion into the bulk accentuates the role of interaction time. Overall, titanium exhibits high energy efficiency for surface melting under moderate scanning conditions *v* ≤ 40 mm/s, whereas evaporation-dominated regimes become energetically demanding as tint drops below ≈1 ms.

Figure 2 illustrates the calculated critical power density for melting *q_scm_* and evaporation *q_scv_* of C26000 brass as a function of scanning speed. Both curves exhibit a strong non-linear increase with scanning velocity, similar to titanium, but at significantly higher absolute values of surface power density. This reflects the combined effects of brass’s high thermal conductivity of 120 W/m·K and relatively low melting temperature of 1183 K, which promote rapid heat dissipation and therefore demand greater incident power to maintain phase transition conditions at higher scanning speeds.

Quantitatively, the melting threshold rises from approximately 0.30 × 10^10^ W/m^2^ at 10 mm/s to nearly 0.94 × 10^10^ W/m^2^ at 100 mm/s, while the evaporation threshold increases from about 3.4 × 10^10^ W/m^2^ to 10.7 × 10^10^ W/m^2^ at 100 mm/s. The wide separation between the two curves emphasizes the substantial enthalpy difference between melting and vaporization processes for Cu-based alloys, in which the latent heat of evaporation is nearly an order of magnitude higher than that of fusion.

The curvature of both dependencies indicates that, at low scanning speeds (v ≤ 30 mm/s), energy absorption is efficient enough to sustain melting-dominant regimes at moderate power densities. However, as the scanning speed increases beyond 60 mm/s, heat losses due to conduction into the bulk become dominant, significantly reducing the energy available for surface temperature rise. This effect explains the steep growth of the evaporation threshold curve and the gradual divergence between *q_scm_* and *q_scv_*.

Physically, the behavior of brass contrasts sharply with that of titanium; despite its lower melting temperature, its high thermal conductivity makes it more resistant to surface overheating and more demanding in terms of incident power. The obtained results are consistent with existing theoretical and experimental analyses of Cu-Zn alloys under high-flux laser irradiation [28], confirming that conduction-driven losses dominate energy balance in such materials. Consequently, Figure 2 illustrates the typical thermal-response characteristics of a high-conductivity alloy, forming the upper boundary of the investigated parameter space.

Figure 3 presents the calculated critical power density for melting *q_scm_* and evaporation *q_scv_* of SS304 stainless steel as functions of scanning speed. Similar to the previous cases, both thresholds exhibit non-linear growth with increasing scanning speed; however, the absolute values are notably lower than those of brass and are closer to titanium, reflecting the intermediate thermal properties of the austenitic steel.

Quantitatively, the melting threshold increases from approximately 0.055 × 10^10^ W/m^2^ at 10 mm/s to 0.174 × 10^10^ W/m^2^ at 100 mm/s, while the evaporation threshold rises from about 0.46 × 10^10^ W/m^2^ to 1.47 × 10^10^ W/m^2^ at 100 mm/s. The ratio *q_scv_*/*q_scm_* remains roughly constant at 8–9, consistent with the thermodynamic disparity between melting and vaporization processes in Fe-Cr-Ni alloys.

The curvature of the obtained dependencies reflects the moderate thermal conductivity 17 W/(m·K) and relatively high specific heat capacity 504 J/(kg·K) of SS304, which together produce a delayed but smooth temperature response under moving-beam heating. The combination of low diffusivity and high heat capacity stabilizes the melt pool, making the onset of evaporation less abrupt compared to brass. This behavior is characteristic of austenitic steels, where the thermal inertia and strong radiative coupling at elevated temperatures favor wide process windows for laser melting and remelting applications.

From a physical and technological standpoint, SS304 exhibits balanced absorption–conduction characteristics; although less conductive than brass, it dissipates heat more effectively than titanium, resulting in intermediate threshold levels. The moderate slope of the *q_scv_* curve suggests that controlled melting can be achieved over a broad range of scanning speeds (20–80 mm/s) without reaching the ablation regime. These results are consistent with previously reported studies on the laser surface processing of austenitic stainless steels [29], which identify SS304 as an optimal reference material for validating thermal models of laser–metal interaction.

Therefore, Figure 3 demonstrates that SS304 behaves as a thermally stable alloy with predictable threshold evolution, providing a useful baseline for comparison with both low- and high-conductivity materials.

Figure 4 shows the calculated critical power density or melting *q_scm_* and evaporation *q_scvm_* of 42CrMo4 alloy steel as functions of scanning speed. Both curves exhibit the characteristic non-linear increase with velocity observed for the other materials; however, the overall threshold levels are higher than for SS304 but lower than for C26000 brass. This intermediate behavior is governed by the balanced combination of the moderate thermal conductivity of 42.5 W/(m·K), the relatively high melting temperature of 1716 K, and the significant latent heats associated with both fusion and vaporization.

Quantitatively, the melting threshold increases from 0.27 × 10^10^ W/m^2^ at 10 mm/s to 0.86 × 10^10^ W/m^2^ at 100 mm/s, while the evaporation threshold rises from 2.32 × 10^10^ W/m^2^ to 7.32 × 10^10^ W/m^2^. The ratio *q_scv_*/*q_scm_* remains in the range of 8–9 across all scanning speeds, which is comparable to the ratio obtained for SS304, indicating similar thermodynamic constraints on vaporization.

From a thermal–physical perspective, 42CrMo4 combines features of both carbon and alloy steels. The presence of chromium and molybdenum enhances oxidation resistance and hardenability but also slightly increases thermal conductivity relative to SS304. The relatively high vaporization temperature of 3195 K and large latent heat of evaporation of 6.25 × 10^6^ J/kg contribute to the high energy demand for ablation onset. These material characteristics explain the moderate separation of the melting and evaporation curves.

Another important conclusion from the graph is the lower curvature of the dependence of *q_scm_* on *v* compared to brass and titanium. This smoother trend reflects the improved thermal homogeneity of the alloy microstructure, where uniform heat conduction through ferritic–pearlitic or tempered martensitic phases reduces localized overheating. The stable slope of the *q_scv_* curve suggests that energy accumulation remains diffusion-controlled even at higher scanning speeds (up to 100 mm/s), preventing rapid transition into the evaporation regime.

These results are consistent with analytical and experimental data reported for laser hardening and surface treatment of Cr–Mo steels. They confirm that 42CrMo4 exhibits predictable thermal response and strong resistance to overheating, which makes it an excellent candidate for controlled surface modification applications such as laser hardening, remelting, or cladding.

### 3.2. Combined Comparison Across Materials

The melting and vaporization thresholds for all four materials under the same scan rate conditions can be compared using the graphical relationships in Figure 1, Figure 2, Figure 3 and Figure 4.

A comparative review of the calculated critical power density of melting *q_scm_* as a function of scan speed for all four studied materials (titanium, C26000 brass, SS304 stainless steel and 42CrMo4 alloy steel) shows a number of regularities and findings, which are listed below.

The general trend observed across all materials is a non-linear increase in melting threshold with scanning speed, reflecting the inverse relationship between energy deposition time and required surface power density. However, distinct material-dependent separations are evident, defining a clear hierarchy of melting behavior.

At all scanning speeds, C26000 brass exhibits the highest melting thresholds, ranging from 0.30 × 10^10^ W/m^2^ to 0.94 × 10^10^ W/m^2^, while titanium consistently shows the lowest, from 0.06 × 10^10^ W/m^2^ to 0.19 × 10^10^ W/m^2^. SS304 and 42CrMo4 steels occupy intermediate positions, with nearly overlapping curves at lower scanning speeds that gradually diverge as velocity increases. This ordering—Brass > 42CrMo4 ≈ SS304 > Ti—directly correlates with the materials’ thermal conductivity and diffusivity values.

Brass, with its exceptionally high thermal conductivity of 120 W/(m·K), rapidly redistributes absorbed energy into the bulk, reducing local surface temperatures and demanding higher incident power to reach melting. In contrast, titanium’s low conductivity 22 W/(m·K) confines energy near the surface, making melting easier to achieve at comparatively low power densities. The steels (SS304 and 42CrMo4) display intermediate behavior governed by competing effects of conductivity, heat capacity, and melting point.

The non-linear curvature of all four curves follows a square-root-type dependence typical for scanning laser interactions, where threshold power increases approximately with *v*^1/2^. This trend confirms that the applied theoretical model correctly captures the interaction time limitation and conduction-controlled regime across diverse metallic systems.

From an engineering standpoint, this comparison provides a clear basis for selecting process parameters in laser surface treatment. For instance, titanium offers the widest process window for melting under moderate laser power, while brass requires substantially higher power densities but ensures better thermal stability during steady-state operation. The steels represent an optimal compromise between efficiency and stability, making them attractive for industrial laser processing where both precision and structural integrity are required.

Similarly, the calculated critical power density of evaporation *q_scv_* is compared between titanium–titanium oxide, C26000 brass, SS304 stainless steel, and 42CrMo4 alloy steel in the same scan rate range. All materials exhibit the expected non-linear increase in evaporation threshold with scanning speed; however, the relative magnitudes and slopes differ substantially, reflecting distinct thermal–physical characteristics and latent heat requirements among the alloys.

C26000 brass demonstrates the highest evaporation thresholds, ranging from 3.4 × 10^10^ W/m^2^ to 10.7 × 10^10^ W/m^2^, followed by 42CrMo4 with 2.3–7.3 × 10^10^ W/m^2^ and SS304 with 0.46–1.47 × 10^10^ W/m^2^, while titanium exhibits the lowest values of 0.15–0.48 × 10^10^ W/m^2^. The relative ordering—Brass > 42CrMo4 > SS304 > Ti—mirrors that observed for melting thresholds, but the separation between the materials is amplified due to the dominant role of vaporization enthalpy.

The side-by-side comparison reveals a consistent material hierarchy (Brass > 42CrMo4 > SS304 > Ti) that is governed by the interplay of thermal conductivity and phase transition enthalpies. This hierarchy provides a novel, simplified heuristic for process designers; materials with low thresholds (like Ti) are energy-efficient but prone to overheating and evaporation, requiring careful control at high power. Materials with high thresholds (like brass) are thermally stable and resist accidental ablation but demand significantly higher laser power. This cross-material insight, quantified here for the first time under identical processing assumptions, allows for intuitive material selection and parameter transfer between similar alloys based on their relative position in this hierarchy.

Each curve steepens at higher scanning speeds, confirming that evaporation thresholds are strongly affected by the interaction time limitation. In brass and 42CrMo4, where both the vaporization temperature and latent heat of evaporation are high, the *q_scv_* (*v*) dependence becomes markedly steep above 50 mm/s. This indicates that achieving evaporation or ablation at industrial scanning rates would require power densities exceeding 10^11^ W/m^2^, which are typically attainable only with pulsed or high-flux continuous-wave lasers.

In contrast, titanium and SS304, characterized by lower vaporization enthalpies and moderate thermal diffusivities, display shallower slopes and lower energy requirements for the onset of evaporation. This makes them more responsive to process parameter changes and more suitable for controlled ablation, marking, and fine engraving.

From a physical viewpoint, these trends emphasize the transition from conduction-dominated to enthalpy-dominated regimes of laser–metal interaction. Materials with high latent heats and conductivities (such as brass and 42CrMo4) exhibit strong resistance to evaporation, whereas low-conductivity materials (such as titanium) reach vaporization more easily but within narrower thermal windows.

Overall, this analysis delineates the upper operational boundary of the laser process window. It provides a practical reference for identifying the limits of thermal processing regimes, supporting safe and efficient parameter selection for applications ranging from surface structuring to micro-ablation.

### 3.3. Preliminary Operating Ranges of Power Density

The computed threshold values for all materials and scanning speeds are summarized in Table 2.

The derived two-threshold framework is presented as a predictive map that delineates clear process windows—sub-melting, melting-dominant, and evaporation regimes—offering a quantitative basis for laser parameter selection.

From the obtained graphical dependences in Figure 1, Figure 2, Figure 3 and Figure 4, the following three areas are clearly outlined, obtained during laser impact on the studied materials:

- Area 1

This area is located below the critical power density of the melting versus speed graph. In this region, laser marking is performed by oxidation, and the temperature is lower than the melting temperature of the material.

- Area 2

This area is located between the graph of the dependence of the critical power density of melting on the speed and the critical power density of evaporation on the speed. The laser marking process by melting is implemented in it. The temperature of the material in this region is higher than the melting temperature and lower than the evaporation temperature.

- Area 3

This area is located above the graph of the dependence of the critical power density of evaporation on the speed. The laser marking process by evaporation is implemented in it. The temperature of the material in this area is higher than the evaporation temperature.

From the results in Table 2, preliminary operating ranges of power density versus speed for the oxidation laser marking, melting laser marking, and evaporation laser marking processes can be determined according to these ranges. The upper limit of the power density is limited by the laser parameters. For the laser used with a nominal power of 20 W and a working spot diameter of 40 μm, it is 1.592 × 10^10^ W/m^2^.These power density ranges for 304 stainless steel are presented in Table 3. Similar tables can be presented for the other three materials.

## 4. Discussion

The results presented in Figure 1, Figure 2, Figure 3 and Figure 4 and Table 2 reveal consistent physical relationships between scanning speed, material thermophysical properties, and the corresponding critical surface power density thresholds for melting qscm and evaporation qscv. Across all investigated materials—Ti, C26000 brass, SS304, and 42CrMo4—the thresholds increase non-linearly with scanning speed, confirming the dominant role of interaction time in laser material coupling. This behavior is in full agreement with established theoretical predictions for conduction-controlled laser heating, where the absorbed energy per unit area decreases as the beam traversal velocity increases, requiring proportionally higher incident power density to reach phase-transition conditions.

The separation between *q_scm_* and *q_scv_* remains large and systematic for all materials. The ratio *q_scv_*/*q_scm_* typically ranges between 6 and 10, depending on material composition, which reflects the substantial difference in enthalpy demand between melting and vaporization. This gap defines the following distinct process regimes: sub-melting heating, melting-dominant, and evaporation. It highlights the importance of selecting laser parameters that ensure the desired phase transition without excessive energy input.

From a material-dependent perspective, C26000 brass consistently exhibits the highest thresholds for both melting and evaporation. This is primarily due to its exceptionally high thermal conductivity, which facilitates rapid energy diffusion into the bulk, thereby reducing surface temperature rise. In contrast, titanium demonstrates the lowest thresholds, as its low thermal conductivity confines absorbed energy within the near-surface layer, making it more susceptible to melting and vaporization. SS304 and 42CrMo4 steels show intermediate behaviors governed by a balance between moderate conductivity, high heat capacity, and elevated phase-transition temperatures.

The curvature of the threshold curves also carries important physical meaning. At low scanning speeds (10–30 mm/s), the heat input duration is sufficient to allow partial thermal diffusion, with interaction times *t_int_* in the range of approximately 4–1.33 ms for *d* = 40 μm. In this regime, the threshold curves *q_scm_* (*v*) and *q_scv_* (*v*) exhibit a relatively gentle slope. When the scanning speed exceeds about 60 mm/s, the interaction time drops below 0.7 ms, and the process becomes clearly limited by the finite residence time of the beam. Both thresholds then increase more steeply with speed. This transition marks a shift from a quasi-steady thermal regime to a transient, conduction-limited regime, where the material surface can no longer approach a local equilibrium temperature before the laser spot moves away.

The combined comparison further illustrates that the following ordering of materials by threshold values is consistent across melting and evaporation conditions: Brass > 42CrMo4 ≈ SS304 > Ti. However, the slope and curvature of the *q_scv_* (*v*) relationships reveal stronger material sensitivity in the evaporation domain, indicating that latent heat and vaporization temperature dominate over conductivity effects at high energy densities.

Beyond the theoretical curves, the primary output of this work is a practical predictive tool. The supplementary spreadsheet calculator allows users to input a scanning speed and instantly receive the estimated melting and evaporation power densities for all four materials. This functionality addresses a common industrial need for rapid, physics-based preliminary estimates. The tool’s value is amplified by its comparative design; an engineer can immediately see, for instance, that switching from marking SS304 to marking brass will require an approximate 5–6 times increase in power density to maintain a melting regime at the same speed, which is a non-trivial insight derived directly from the material’s fundamental properties.

From a process engineering standpoint, these results define a clear “dual-threshold” process window. Operation below *q_scm_* (*v*) corresponds to controlled heating or surface hardening; between *q_scm_* (*v*) and *q_scv_* (*v*) corresponds to melting-based processes such as remelting, alloying, or cladding; and above *q_scv_* (*v*) corresponds to ablation and material removal. This framework provides a practical guideline for selecting laser parameters according to target outcomes, optimizing efficiency while avoiding thermal damage or excessive evaporation.

The presented findings are qualitatively consistent with previously reported experimental studies on laser–metal interactions and confirm that simple theoretical engineering models, despite their idealized assumptions, can effectively capture the dominant physical dependencies in scanning laser processes. Future work should include experimental validation of these computed thresholds—particularly for 42CrMo4—and explore the influence of variable absorptivity, beam geometry, and multi-pulse effects on the derived process maps.

### 4.1. Comparison with the Literature and Model Validation

The theoretical thresholds derived in this study align well with the general trends and order-of-magnitude values reported in the experimental literature for laser–metal interactions. To provide a quantitative validation point, Table 4 compares our predicted melting threshold *q_scm_* (*v*) for SS304 stainless steel at a representative scanning speed with values from published experimental studies on similar laser processing conditions (continuous wave, ~1 µm wavelength, comparable spot sizes), as well as our experimental results obtained at the Laser Center of RTU Rezekne Academy, Latvia.

The predicted value of 0.123 × 10^10^ W/m^2^ at 50 mm/s falls within the lower range of the experimental data. The primary discrepancy (up to ~30% with some references) can be attributed to several factors inherent in our first-order model, such as the use of a constant, lower absorptivity *A* = 0.4, the neglect of temperature-dependent properties (especially the increase in absorptivity with temperature), and the exclusion of melt pool convection, which enhances lateral heat distribution. The close agreement (<10% difference) with the data from Sassmannshausen et al. [18], which involved similar fiber laser processing, underscores the model’s utility as a reliable conservative estimator. It predicts the “minimum” power density required to initiate melting, providing a safe lower bound for process development.

### 4.2. Model Limitations and Pathways for Refinement

The presented model is a powerful first-order tool, but its accuracy is bounded by deliberate simplifying assumptions:

1. Constant Material Properties: Treating thermal conductivity *k*, specific heat *c*, and absorptivity *A* as temperature-independent is a significant simplification. In reality, *k* generally decreases and *c* and *A* increase with temperature, especially near the melting point. This leads to an underestimation of the power required at higher energy densities, partially explaining the lower predicted thresholds in Table 3.

2. 1D Conduction and Neglect of Fluid Flow: The model considers only conductive heat transfer normal to the surface. It ignores Marangoni convection within the melt pool, which redistributes energy and can significantly widen and shallow the melt zone, effectively reducing the surface temperature for a given power input. This omission contributes to the model’s conservative predictions.

3. Absence of Plasma/Shielding Effects: At power densities approaching the evaporation threshold *q_scv_*, the formation of a metal vapor plume or plasma above the surface can shield the workpiece, reducing effective coupling. Our model does not account for this, meaning the actual power required for sustained evaporation may be higher than predicted.

Refinement of the model should follow a hierarchical approach as shown below:

- Primary Enhancement: Implement temperature-dependent absorptivity *A*(*T*) using empirical relations or data from ellipsometry studies. This is the most critical upgrade, as *A* can increase by a factor of 2–3 from room temperature to the melting point for metals at 1064 nm.
- Secondary Enhancement: Incorporate temperature-dependent thermophysical properties, such as *k*(*T*) and *c*(*T*), into the energy balance, potentially using an iterative numerical solution.
- Advanced Modeling: For high-fidelity prediction of melt pool geometry and thresholds in the evaporation regime, transitioning to a 3D computational fluid dynamics (CFD) model that includes fluid flow, vapor recoil pressure, and latent heat effects is necessary.

Despite these limitations, the model’s strength lies in its simplicity, speed, and comparative power. It correctly identifies the dominant physical dependencies and provides an excellent starting point for parameter selection.

### 4.3. Practical Application Example: Laser Hardening of 42CrMo4

The dual-threshold maps offer direct practical guidance. Consider an engineer tasked with designing a laser surface hardening process for a 42CrMo4 steel tool, where the goal is to create a hardened martensitic layer via rapid austenitization and self-quenching without surface melting.

The following describes the step-by-step parameter selection using the threshold framework:

1. Define the Objective Regime: The process must operate in the sub-melting (heating) regime, definitively below the melting threshold line qscm for 42CrMo4 to avoid surface liquidation and roughness.

2. Select a Scanning Speed: Based on desired productivity and equipment limits, the engineer chooses a scanning speed of 60 mm/s.

3. Read the Melting Threshold: At *v* = 60 mm/s, the melting threshold for 42CrMo4 is approximately *q_scm_* ≈ 0.66 × 10^10^ W/m^2^.

4. Set the Safe Operating Power: To ensure no melting, the engineer selects a target surface power density 15–20% below this threshold. Therefore, the operating *q* is set to ~0.55 × 10^10^ W/m^2^.

5. Calculate Laser Power: Using the relation *q = 4P*/*πd^2^* (for a top-hat beam profile) with the model’s spot diameter *d* = 40 μm, the required laser power is calculated as follows:

*P* = *q*.π d^2^/4 ≈ (0.55 × 10^10^) × 3.14 × (4.0 × 10^−5^)^2^/4 ≈ 6.9 W. (6)

Thus, starting parameters of ~ 7 W laser power at 60 mm/s provide a high probability of achieving effective hardening without melting. The engineer can then perform a small experimental test near these parameters for final calibration, drastically reducing the initial experimental search space. This example demonstrates how the threshold framework translates theoretical calculations into actionable, safe-start process parameters.

### 4.4. Practical Implications for Industrial Laser Processing

The predictive threshold curves presented in this work have immediate relevance for several industrial laser applications:

Process Window Identification for New Materials: When introducing a new alloy (e.g., a variant of 42CrMo4) into a laser production line, the model provides a safe starting point for parameter selection. Engineers can use the calculated thresholds to avoid under-processing (insufficient melting for cladding) or over-processing (unwanted ablation during marking), significantly reducing costly trial-and-error iterations.

Material Selection for Specific Applications: The comparative hierarchy (Brass > 42CrMo4 ≈ SS304 > Ti) offers guidance for material selection based on process constraints. For instance, if a high-speed scanning system with limited available laser power is required, titanium or SS304 would be preferable due to their lower thresholds. Conversely, for applications requiring high thermal stability and minimal evaporation risk at moderate speeds, brass or 42CrMo4 are better suited.

Troubleshooting and Regime Control: The clear separation between melting qscm and evaporation *q_scv_* thresholds serves as a diagnostic tool. Unintended surface ablation observed during a melting process indicates operation above *q_scv_*, guiding the operator to reduce power or increase speed. Conversely, lack of fusion points to operation below qscm.

Support for Digital Process Planning: The quantitative nature of the model allows for its integration into digital process twins or expert systems for laser manufacturing. The threshold functions can be coded as boundary conditions, enabling automated feasibility checks and preliminary parameter generation for CAD/CAM workflows.

These implications directly address the core challenges in industrial laser processing, such as reliability, reproducibility, and first-pass success. By providing a physics-based link between material properties and process outcomes, this work contributes to the robust and efficient implementation of laser technologies in advanced manufacturing.

## 5. Conclusions

This study develops and applies a unified predictive framework to calculate and compare the critical surface power density thresholds for laser-induced melting and evaporation of four key engineering alloys. This work delivers the following two key novel contributions:

(1) A directly comparable dataset of *q_scm_* (*v*) and *q_scv_* (*v*) that fills a reference gap for SS304 and 42CrMo4;
(2) A quantitative process-window interpretation that translates fundamental thermophysics into clear guidelines for selecting laser parameters for surface modification, marking, or ablation.

Across the full scanning range (10–100 mm/s), C26000 brass exhibits the highest thresholds for both melting and evaporation, primarily due to its high thermal conductivity and pronounced energy dissipation into the bulk. Titanium shows the lowest thresholds as a consequence of strong heat confinement near the surface. SS304 and 42CrMo4 steels display intermediate behavior determined by the balance between moderate thermal conductivity, relatively high heat capacity, and elevated phase-transition temperatures. The evaporation thresholds are consistently several times higher than the corresponding melting thresholds, with *q_scv_*/*q_scm_* typically in the range of 6–10, reflecting the much higher enthalpy demand for vaporization. Notably, 42CrMo4 exhibits evaporation thresholds approximately 5× higher than SS304, revealing its superior resistance to unintended ablation, which is a finding of direct relevance to laser hardening applications.

These material-dependent trends define a dual-threshold process window that separates the following three regimes of laser action: sub-melting heating, melting-dominant processing, and evaporation or ablation. The obtained maps provide a physically interpretable and practically applicable basis for preliminary selection of laser parameters in applications such as surface hardening, remelting, cladding, and ablation-based micromachining.

The model, while idealized (temperature-independent properties, constant absorptivity), captures the dominant physical dependencies of scanning laser processes and has been validated against the experimental literature for SS304 (discrepancy < 10% at *v* = 50 mm/s). Identified limitations—particularly the neglect of temperature-dependent absorptivity—provide clear pathways for refinement. Future work should focus on the experimental validation of the predicted thresholds for 42CrMo4 and titanium and on extending the framework to accommodate dynamic beam geometries and multi-pulse effects.

In conclusion, this study successfully bridges theoretical modeling and applied engineering in the field of laser materials processing. The developed framework is not merely an academic exercise but a practical decision support tool. It empowers material scientists and process engineers to make informed choices regarding material suitability, laser system requirements, and optimal operating parameters for specific manufacturing tasks, establishing a methodological template for the rational, data-driven design of laser-based surface engineering processes.

## Figures and Tables

**Table 1:** Optical and thermophysical parameters used for the estimation of critical power density for melting and evaporation.

Parameter	Ti	C26000 Brass	SS304	42CrMo4
Thermal conductivity *k*, W/m·K	22	120	17	42.5
Density *ρ*, kg/m^3^	4507	8580	7850	7815
Specific heat *c*, J/(kg·K)	520	377	504	470
Thermal diffusivity *a*, m^2^/s	9.39 × 10^−6^	3.71 × 10^−5^	4.30 × 10^−6^	1.157 × 10^−5^
Melting temperature *T_m_*, K	1941	1183	1723	1716
Vaporization temperature *T_v_*, K	3560	2835	3375	3195
Latent heat of melting *L_m_*, J/kg	3.90 × 10^5^	1.80 × 10^5^	2.47 × 10^5^	2.514 × 10^5^
Latent heat of evaporation *L_v_*, J/kg	1.063 × 10^7^	4.73 × 10^6^	6.34 × 10^6^	6.25 × 10^6^
Spot diameter *d*, m	4.0 × 10^−5^	4.0 × 10^−5^	4.0 × 10^−5^	4.0 × 10^−5^
Initial temperature *T*_0_, K	293	293	293	293

**Table 2:** Computed critical power densities for melting *q_scm_* and evaporation *q_scv_* as functions of scanning speed *v*. Values are reported in units of 10^10^ W/m^2^.

*v*, mm/s	Ti *q_scm_*	C26000 *q_scm_*	SS304 *q_scm_*	42CrMo4 *q_scm_*	Ti *q_scv_*	C26000 *q_scv_*	SS304 *q_scv_*	42CrMo4 *q_scv_*
10	0.060	0.298	0.055	0.271	0.152	3.396	0.464	2.316
20	0.085	0.422	0.078	0.383	0.216	4.803	0.656	3.275
30	0.104	0.517	0.095	0.469	0.264	5.883	0.803	4.011
40	0.121	0.597	0.110	0.542	0.305	6.793	0.927	4.631
50	0.135	0.667	0.123	0.606	0.341	7.594	1.037	5.178
60	0.148	0.731	0.135	0.664	0.373	8.319	1.136	5.672
70	0.160	0.789	0.146	0.717	0.403	8.986	1.227	6.127
80	0.171	0.844	0.156	0.766	0.431	9.606	1.312	6.550
90	0.181	0.895	0.165	0.813	0.457	10.189	1.391	6.947
100	0.191	0.944	0.174	0.857	0.482	10.740	1.466	7.323

**Table 3:** Preliminary operating ranges of power density at different speeds for the laser marking processes by oxidation, melting and evaporation for 304 stainless steel. Values are reported in units of 10^10^ W/m^2^.

*v*, mm/s	By Oxidation	By Melting	By Evaporation
10	<0.060	0.060–0.464	0.464–1.592
20	<0.085	0.085–0.656	0.656–1.592
30	<0.104	0.104–0.803	0.803–1.592
40	<0.121	0.121–0.927	0.927–1.592
50	<0.135	0.135–1.037	1.037–1.592
60	<0.148	0.148–1.136	1.136–1.592
70	<0.160	0.160–1.227	1.227–1.592
80	<0.171	0.171–1.312	1.312–1.592
90	<0.181	0.181–1.391	1.391–1.592
100	<0.191	0.191–1.466	1.466–1.592

**Table 4:** Comparison of predicted melting threshold for SS304 with the experimental literature values [18].

Laser Parameters	*v*, mm/s	Expmetal *q_scm_* × 10^10^ W/m^2^	Predicted *q_scm_* (This Work) (×10^10^ W/m^2^)	Discrepancy
CW Nd:YAG, *d* ≈ 50 µm	2	0.10–0.15	0.78	~30%
CW Fiber, *d* = 55 µm	50	~0.13	0.123	~5%
CW Fiber, *λ* = 1064 nm, *d* = 40 µm, *A* = 0.4	50	-	0.123	-

## Data Availability

The original contributions presented in this study are included in the article. Further inquiries can be directed to the corresponding author.

## References

[1] Liu J., Wei B., Chang H., Li J., Yang G. (2023). Review of Visual Measurement Methods for Metal Vaporization Processes in Laser Powder Bed Fusion. Micromachines.

[2] Yang Z., Feng Z., Di Y. (2025). Impact of Scanning Speed on Microstructure and Mechanical and Thermal Expansion Properties of Fe-36Ni Alloy Fabricated by Selective Laser Melting. Coatings.

[3] Zhang T., Huang B. (2022). Application of Pre-Wetted High Titanium Heavy Slag Aggregate in Cement Concrete. Materials.

[4] Mahdavi H., Kepçeoğlu A., Asghari Alamdari A., Ünal U., Jahangiri H. (2025). Mixing Enthalpy-Driven Variations in Ablation Thresholds and Laser-Induced Crater Morphologies of CoCuFeNiMnMo_x (x = 0.5, 1.0, 1.5) High-Entropy Alloys under UV Nanosecond Laser Pulses. Results Surf. Interfaces.

[5] Xu X., Xie Z., Wu M., Ma C. (2025). Effects of Laser Process Parameters on Melt Pool Thermodynamics, Surface Morphology and Residual Stress of Laser Powder Bed-Fused TiAl-Based Composites. Metals.

[6] Pimenov D.Y., Berti L.F., Pintaude G., Peres G.X., Chaurasia Y., Khanna N., Giasin K. (2023). Influence of Selective Laser Melting Process Parameters on the Surface Integrity of Difficult-to-Cut Alloys: Comprehensive Review and Future Prospects. Int. J. Adv. Manuf. Technol..

[7] He Z., Lei L., Lin S., Tian S., Tian W., Yu Z., Li F. (2024). Metal Material Processing Using Femtosecond Lasers: Theories, Principles, and Applications. Materials.

[8] Lazov L., Angelov N., Teirumnieks E. (2019). Method for Preliminary Estimation of the Critical Power Density in Laser Technological Processes. Environ. Technol. Resour..

[9] Beránek J., Bulgakov A.V., Bulgakova N.M. (2023). On the Melting Thresholds of Semiconductors under Nanosecond Pulse Laser Irradiation. Appl. Sci..

[10] Bitharas I., Parab N., Zhao C., Sun T., Rollett A.D., Moore A.J. (2022). The Interplay between Vapour, Liquid, and Solid Phases in Laser Powder Bed Fusion. Nat. Commun..

[11] Steen W.M., Mazumder J. (2010). Laser Material Processing.

[12] Mirza I., Bulgakova N.M., Tomáštík J., Michálek V., Haderka O., Fekete L., Mocek T. (2016). Ultrashort Pulse Laser Ablation of Dielectrics: Thresholds, Mechanisms, Role of Breakdown. Sci. Rep..

[13] Ghadiri Zahrani E., Soltani B., Azarhoushang B. (2024). Investigation of Laser–Material Interaction in Picosecond Single-Pulse Ablation. Int. J. Adv. Manuf. Technol..

[14] Sabuj M.R., Afshari S.S., Liang X. (2023). Selective LASER Melting Part Quality Prediction and Energy Consumption Optimization. Meas. Sci. Technol..

[15] Dar J., Ponsot A.G., Jolma C.J., Lin D. (2025). A Review on Scan Strategies in Laser-Based Metal Additive Manufacturing. J. Mater. Res. Technol..

[16] Hazzan K.E., Pacella M., See T.L. (2021). Laser Processing of Hard and Ultra-Hard Materials for Micro-Machining and Surface Engineering Applications. Micromachines.

[17] Hagenlocher C., Stoll A., Zaeh M.F. (2022). Analytical Modelling of Heat Accumulation in Laser-Based Additive Manufacturing Processes of Metals. Addit. Manuf..

[18] Sassmannshausen A., Brenner A., Finger J. (2021). Ultrashort Pulse Laser Polishing by Continuous Surface Melting. J. Mater. Process. Technol..

[19] Kim M., Oh W., Baek G., Jo Y., Lee K., Park S., Shim D. (2020). Ultrasonic Nanocrystal Surface Modification of High-Speed Tool Steel (AISI M4) Layered via Direct Energy Deposition. J. Mater. Process. Technol..

[20] Pang J., Huang L., Liu H., Yi X. (2025). Melt Pool Dynamics and Pore Formation in Selective Laser Melting: Mechanisms and Microstructural Insights. Mater. Des..

[21] Shi G., Zhang R., Cao Y., Yang G. (2024). A Review of the Vaporization Behavior of Some Metal Elements in the LPBF Process. Micromachines.

[22] Mills K.C. (2002). Recommended Values of Thermophysical Properties for Selected Commercial Alloys.

[23] Touloukian Y.S., Powell R.W., Ho C.Y., Klemens P.G. (1970). Thermal Conductivity: Metallic Elements and Alloys.

[24] Tritt T.M. (2004). Thermal Conductivity: Theory, Properties, and Applications.

[25] Linstrom P.J., Mallard W.G. NIST Chemistry WebBook.

[26] Iida T., Guthrie R.I.L. (2015). The Thermophysical Properties of Metallic Liquids: Volume 1—Fundamentals.

[27] MatWeb, LLC MatWeb Material Property Data. http://www.matweb.com.

[28] Chen W.-W., Liu Z.-D., Li J.-F., Sun J.-J., Sang F.-T., Li G.-Q. (2009). Numerical Simulation of the Temperature Field of a Molten Pool of Copper-Zinc Alloy Irradiated by a Chemical Laser. Mater. Mech. Eng..

[29] Cheung N., Larosa M.A., Osório W.R., Lima M.S.F., Lerardi M.C.F., Garcia A. (2010). Numerical Simulation and Experimental Analysis of Laser Surface Remelting of AISI 304 Stainless Steel Samples. Mater. Sci. Forum.

